# Fate of patients with slipped capital femoral epiphysis (SCFE) in later life: risk of obesity, hypothyroidism, and death in 2,564 patients with SCFE compared with 25,638 controls

**DOI:** 10.1080/17453674.2020.1749810

**Published:** 2020-04-14

**Authors:** Yasmin D Hailer

**Affiliations:** Section of Orthopedics, Department of Surgical Sciences, Uppsala University, Sweden

## Abstract

Background and purpose — Associations between obesity and slipped capital femoral epiphysis (SCFE) during adolescence are described; however, few studies report on the lifetime risk of obesity in patients with SCFE. In addition, with the obesity epidemic in children and adolescents, an increasing incidence of SCFE might be expected. An association of SCFE with hypothyroidism seems ambiguous, and the association between SCFE and depression and all-cause mortality has not yet been evaluated. This study investigates the associations of SCFE with obesity, hypothyroidism, depression, and mortality, and putative changes in the yearly incidence of SCFE.

Patients and methods — 2,564 patients diagnosed with SCFE at age 5–16 diagnosed between 1964 and 2011 were identified in the Swedish Patient Register. These were matched for age, sex, and residency with unexposed control individuals. Cox regression models were fitted to estimate the risk of obesity, hypothyroidism, depression, and death, in exposed compared with unexposed individuals.

Results — The risk of obesity (HR 9, 95% CI 7–11) and hypothyroidism (HR 3, CI 2–4) was higher in SCFE patients compared with controls. There was no increase in the risk of developing depression (HR 1, CI 1–1.3) in SCFE patients. In contrast, all-cause mortality was higher in SCFE patients than in controls (HR 2, CI 1–2). The incidence of SCFE did not increase over the past decades.

Interpretation — Patients with SCFE have a higher lifetime risk of obesity and hypothyroidism and a higher risk of all-cause mortality compared with individuals without SCFE. These findings highlight the lifetime comorbidity burden of patients who develop SCFE in childhood, and increased surveillance of patients with a history of SCFE may be warranted. The incidence of SCFE did not increase over the last decades despite increasing obesity rates.

Slipped capital femoral epiphysis (SCFE) occurs commonly in overweight children and adolescents. The etiology of the disease is still unknown but several studies have concluded that overweight and obesity are catalyzing factors, either by overloading the growth plate (Fishkin et al. 2006) or as an endocrine condition diminishing the stability of the growth plate. The latter would explain the age-dependent relationship between obesity and SCFE onset, where obese children are found to suffer from SCFE at a younger age compared with children of age- and length-adequate weight (Perry et al. 2018). Wensaas et al. (2011) investigated the long-term outcome of 66 patients with a history of SCFE and found that one-third were overweight or obese in adulthood. However, the risk of developing obesity in SCFE patients, not only in childhood but during later life, is still unknown.

In contrast, presuming obesity as a causal factor, one would expect higher incidences of SCFE due to epidemic obesity rates in children and adolescents (Murray and Wilson 2008). However, comparisons of incidence rates are often difficult because the calculations are based on different age groups and changes in incidence rates over the past decades have not yet been reported.

Inconsistent findings concerning the association between SCFE and hypothyroidism have emerged. Some authors found no association between SCFE and hypothyroidism (Brenkel et al. 1988), whereas others found an association of the 2 diseases (Kadowaki et al. 2017). Congenital hypothyroidism is part of the screening program of newborns in Sweden (National Board of Health and Welfare 2018) but acquired hypothyroidism is often underdiagnosed in children and adolescents (Ghaemi et al. 2015). To my knowledge, there is no study investigating the lifetime risk of hypothyroidism in patients with a history of SCFE.

Studies focusing on the long-term outcome after SCFE (Wensaas et al. 2011, Castaneda et al. 2013, Wiemann and Herrera-Soto 2013, de Poorter et al. 2016) attest that SCFE is not only a childhood hip disease: In some patients SCFE transforms into a chronic disease by creating hip joint impingement (Lerch et al. 2019) or premature osteoarthritis, or both (Goodman et al. 1997). It is known that patients with chronic diseases are at greater risk of developing depression (Moussavi et al. 2007, Podeszwa et al. 2015) and die earlier (Ng et al. 2007, Hailer and Nilsson 2014) compared with the general population.

Thus, the lifetime burden of obesity, hypothyroidism, and depression in patients exposed to SCFE remains unclear and leads to the following questions: (1) Do patients with SCFE have an increased lifetime risk of obesity and hypothyroidism? (2) Has the average incidence of SCFE in Sweden changed over the past few decades? (3) Is SCFE associated with a higher risk of depression and a higher risk of all-cause mortality?

## Patients and methods

### Study design and data sources

To conduct this nationwide, population-based cohort study the Swedish Patient Register was used to identify all patients with a diagnosis of SCFE (ICD-7: 732.03, ICD-8: 722.10, ICD-9: 732.2, ICD-10: M93.0) from 1964 (when the register was established) until 2011. Through the Swedish Total Population Register, up to 10 controls without SCFE were matched, using the matching criteria date of birth, sex, region of residence, and being alive at the time of SCFE diagnosis, to compare the risk of obesity (ICD-7: 287.09, ICD-8: 277.99, ICD-9: 278 and 783.6, ICD-10: E66.0), hypothyroidism (ICD-7: 250–252, ICD-8: 240–242, ICD-9: 240–244, ICD-10: E00–E03) and depression (ICD-7: 790.29, ICD-8: 790.20, ICD-9: 308 and 311, ICD-10: F92.0 and F32-38). The Swedish Patient Register provides information on diagnosis codes and dates of admissions and discharge for all individuals in Sweden. Whenever hospital care (in- or outpatient) is given, it is mandatory for all public and private hospitals to deliver information on dates of admission and discharge, registered diagnoses (categorized by the International Classification of Diseases [ICD]), and applied treatments to the Swedish Patient Register together with the unique personal identification number of each individual. Primary health care institutions do not report to the Swedish Patient Register. However, whenever the SCFE diagnosis is apparent on radiographs the patient is referred to hospital care (in- or outpatient). The control group was identified through the Swedish Population Register based on the matching criterion. Statistics Sweden, a government agency, provided information on population size and changes, such as the number of births, deaths, and immigration and emigration at time of interest.

### Study population

The study population was followed from 1964 until the diagnosis of interest (obesity, hypothyroidism, depression), death, emigration, or December 31, 2013, whichever occurred first. The endpoints were obesity (based on the World Health Organization [WHO] criterion of body mass index (BMI) ≥ 30), hypothyroidism, depression, and death from all causes. Any given individual could experience multiple endpoints when diagnosed with more than one diagnosis of interest.

The initial database contained 2,989 patients diagnosed with SCFE and 29,876 controls. 11 SCFE patients were excluded because no matching controls were found. In addition, patients whose age at SCFE diagnosis was < 5 years or ≥ 17 years, those with diagnosed developmental dysplasia (7 patients) of the hip, and those with Legg–Calvé–Perthes disease (38 patients) were excluded together with their controls.

### Characteristics of the study population

**Figure 1.**
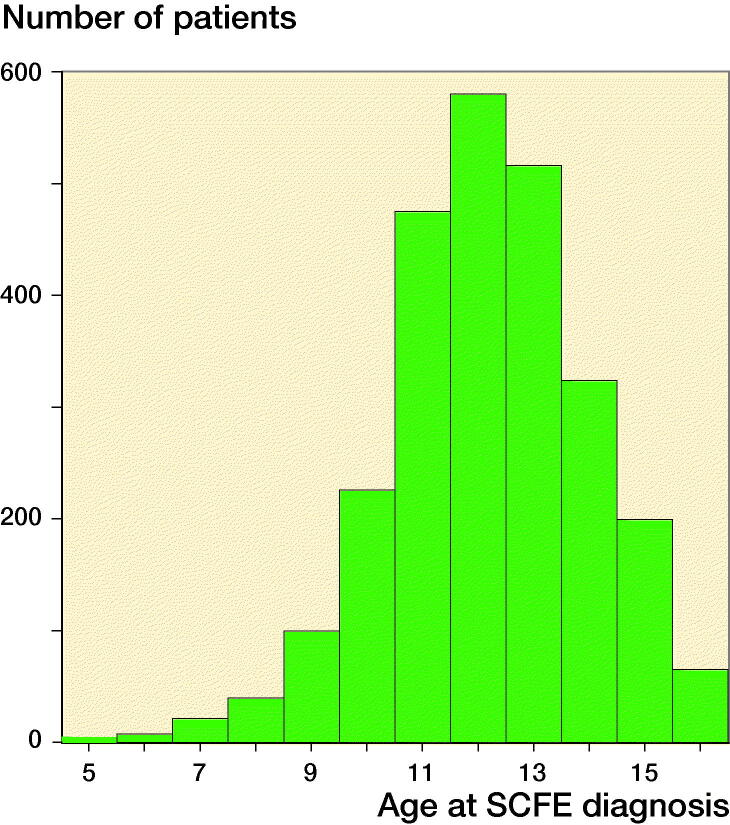
Age at onset of SCFE diagnosis with a peak between 10 and 15 years of age.

The final study population consisted of 2,564 SCFE patients and 25,638 matched controls; there were 11,253 (40%) females, and the mean follow-up time was 34 years (5–66). Median year of birth was 1980 (range 1948–2004), and the mean age at SCFE onset was 12.7 years (Figure 1).

### Statistics

Kaplan–Meier analysis was used to calculate cumulative unadjusted survival functions, with each of comorbidity or death as separate endpoints. Cox proportional hazard models were fitted to estimate the hazard ratio (HR) of developing obesity, hypothyroidism, depression, and death in patients with SCFE compared with unexposed individuals, with or without adjustment for birth year and sex. The assumption of proportional hazards was verified by visual inspection of unadjusted cumulative survival function plots. Estimation uncertainty was assessed by calculating 95% confidence intervals (CIs). Stratified analyses were performed by sex and by the different ICD coding periods described earlier (data not shown). The annual incidence density for SCFE between 1977 and 2011 was calculated based on the population data provided by Statistic Sweden for each year of interest. We calculated the incidence density for SCFE by dividing the number of incident cases per year versus the number of person-years at risk for each corresponding year. We approximated the number of person-years at risk by calculating the average of the population size at risk (children between age 5 and 16 years resident in Sweden) at the start and end of each year of interest. To calculate the 95% CI for the incidence density, the standard deviation (SD) is estimated as sqrt((p × (1–p))/N) and the 95% CI is estimated as p ±1.96 × SD. In order to make this study comparable to the study group of Herngren et al. (2017) a sub-group analysis for SCFE patients with a narrower and more typical age frame for SCFE was performed. Only patients with SCFE diagnosis at age 9 and 15 years between 2000 and 2006 were analyzed.

All statistical analyses were performed using R statistical software (Version 3.3.3; R Foundation for Statistical Computing, Vienna, Austria), including the rms, magrittr, Gmisc, ggplot2, Formula and MASS packages.

### Ethics, funding, data sharing, and potential conflicts of interest

This study was approved by the Ethics Research Committee in Uppsala, Sweden (registration number 2012/065, date of issue March 21, 2012). This research was not supported by grants from any funding agency in the public, commercial or not-for-profit sectors. The dataset that is necessary to replicate main findings can be obtained from the author upon reasonable request. I have no conflicts of interest to declare.

## ^Results^

### Lifetime risk of comorbidities and death in SCFE patients compared with unexposed individuals

The risk of developing obesity was higher in patients with SCFE than in controls (HR 9, CI 7–11), as was the risk of developing hypothyroidism (HR 3, CI 2–4). The risk of developing obesity was higher in male (HR 11, CI 8–16) than in female SCFE patients (HR 7, CI 5–10), and a similar pattern was seen for the risk of hypothyroidism, which was higher in male (HR 5, CI 3–8) than in female SCFE patients (HR 2, CI 2–4; Table 1).

**Table 1.** Prevalence of obesity, hypothyroidism, depression, or death in patients with SCFE and in controls. Values are number (%)

	Control	SCFE
Comorbidities	n = 25,638	n = 2,564
Obesity	129 (0.5)	109 (4.3)
Hypothyroidism	166 (0.7)	46 (1.8)
Depression	998 (3.9)	101 (3.9)
Death	329 (1.3)	52 (2.0)

There was no statistically significant difference in the risk of depression between the SCFE patients and controls (HR 1, CI 1–1.3). During the observation period, 381 (1.4%) individuals died. There were 52 deaths in the SCFE group with the youngest dying at the age of 9 years and the oldest at the age of 53 years. 329 were dead in the control group with the youngest dying at the age of 8 months and the oldest dying at the age of 63 years. The all-cause mortality risk was higher in SCFE patients than in controls (HR 2, CI 1–2). Adjustment for birth year and sex hardly changed the estimates (Table 2). The causes of death are given in Table 3.

**Table 3.** Causes of death in patients with SCFE and in controls. Values are number (%)

	Control	SCFE
Cause of death	n = 25,638	n = 2,564
Vascular	38 (0.2)	14 (0.6)
Endocrine	5 (0.02)	5 (0.2)
Injury	70 (0.3)	8 (0.3)
Neuropsychiatric	15 (0.1)	3 (0.1)
Cancer	66 (0.3)	4 (0.2)
Suicide	36 (0.1)	4 (0.2)

**Table 2.** Hazard risks (HR) with 95% confidence intervals (CI) of comorbidities and all-cause mortality in patients with SCFE and in controls

Comorbidities	HR (CI)	aHR (CI) **^a^**
Obesity	8.7 (6.7–11.2)	8.7 (6.7–11.2)
Hypothyroidism	2.8 (2.0–3.9)	2.8 (2.0–3.9)
Depression	1.0 (0.8–1.3)	1.0 (0.8–1.3)
Death	1.6 (1.2–2.1)	1.6 (1.2–2.1)

**^a^** Adjustment for birth year and sex.

### Incidence density of SCFE in Sweden over the past decades

**Figure 2.**
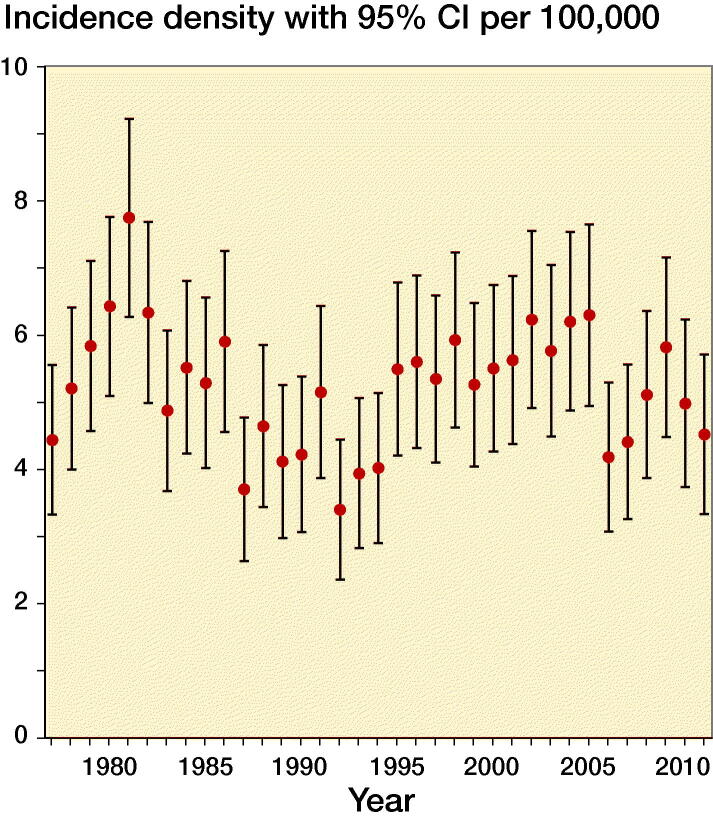
Incidence density of SCFE diagnoses per 100,000 children aged 5 to 16 years in Sweden from 1977 to 2011.

The annual incidence density of SCFE varied from 3.4/100,000 to 7.8/100,000 in children aged 5 to 16 years between 1977 and 2011 (Figure 2). In order to make these data comparable with those calculated by Herngren et al. (2017) a subgroup analysis of children aged 9 to 15 years between 2000 and 2006 was performed. In this 7-year-period 529 children were diagnosed with SCFE. The average annual number of children at risk was 833,632.

### Temporal sequence of SCFE and comorbidities

**Figure 3.**
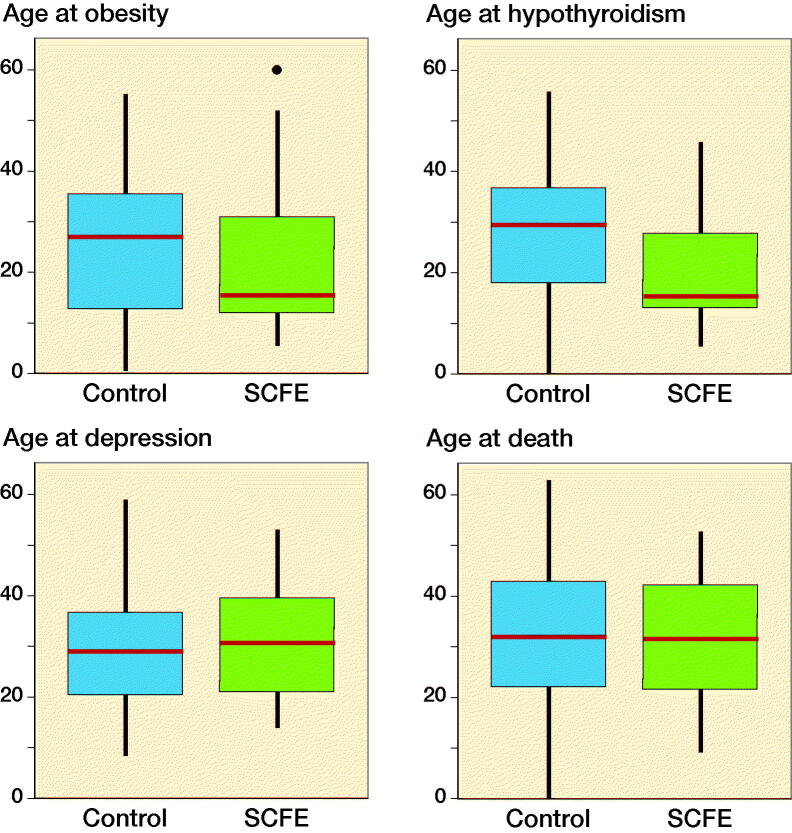
Age at onset of obesity, hypothyroidism, depression, or death of SCFE patients. Age at onset of the diagnosis of obesity and hypothyroidism is younger in patients with SCFE than in the control group (obesity: p-value < 0.05; hypothyroidism: p-value < 0.001). No differences were found for age at diagnosis of depression or death between the two groups. The boxes represent the interquartile range and the red line median. The whisker represents the age range at diagnosis or death.The dot represents an outlier.

In most patients the comorbidity diagnosis was registered after the SCFE diagnosis. Only 20 patients were diagnosed with obesity (1 to 7 years) before being diagnosed with SCFE, and 5 were diagnosed with hypothyroidism (a few months to 7 years) before being diagnosed with SCFE. None had a diagnosis of depression before the SCFE diagnosis. The mean and median age at diagnosis for the comorbidities obesity and hypothyroidism was lower in patients with SCFE than in the control group (Figure 3).

A sensitivity analysis involving only patients and controls who were registered from 2001 when both in- and outpatient hospital care were registered revealed that the adjusted risks for obesity (HR 6, CI 3–10) and hypothyroidism (HR 3, CI 2–8) remained higher in SCFE patients than in controls. In this subgroup, the risk of developing depression was similar for SCFE patients and controls.

## Discussion

SCFE is one of the most common hip disorders in children that can lead to lifelong impairment of hip function. Associations between SCFE and obesity and hypothyroidism have been described but many studies used a retrospective design and small cohorts (Bhatia et al. 2006, Ucpunar et al. 2018). In the past years reports from national databases have increased (Murray and Wilson 2008, Herngren et al. 2017, Perry et al. 2017, Ravinsky et al. 2019), making it possible to analyze epidemiological characteristics of different disease conditions. However, studies emerging from these databases lack a control group or have only appeared in the past decade, making it difficult to make observations before and after exposure or to analyze changes of incidences over time. Moreover, I know of no population-based study describing the lifetime comorbidities of patients with SCFE compared with those without SCFE that focuses on obesity, hypothyroidism, depression, and mortality.

This study finds that patients with a history of SCFE have a 9-fold higher risk of being diagnosed with obesity than their age- and sex-matched controls, with a higher risk for male patients. Over time, the prevalence of obesity in children has increased in both sexes (Eriksson et al. 2018). Perry et al. (2018) found a strong association of BMI and SCFE, with a higher risk for SCFE in children with obesity and high BMI compared with children of normal BMI.

The concerns regarding the rising incidence of SCFE relative to the increasing prevalence of obesity in children (Neovius et al. 2006, Nguyen et al. 2011) are not supported by this study. As a matter of fact, the incidence rate has some variation since 1978, with peaks in 1981 and 2002, but it then remains roughly the same until 2011. In comparison, Herngren et al. (2017) investigated the incidence of SCFE in children between 9 and 15 years old for the period 2007 to 2013 and identified 379 children with SCFE. The average annual number of children 9–15 years old in Sweden between 2007 and 2013 was 726,304. We analyzed a comparable preceding 7-year-period from 2000 to 2006 with an average annual number of children at risk of 833,632 and identified 529 children with SCFE. Thus, the incidences in these two 7-years periods were similar. Interestingly, a 20-year periodicity of peak incidences has also been described for the period from 1900 to 1970 (Hagglund et al. 1984). Nevertheless, the underlying causes of these long-term fluctuations are unclear.

The inconsistency between rising obesity rates in children, on the one hand, and stable rates for SCFE, on the other, together with the elevated risk of developing obesity in later life found in this study indicates that not only obesity per se but other factors associated with obesity may play a role in the etiology of SCFE. Halverson et al. (2017) reported elevated serum leptin levels in patients with SCFE, regardless of their BMI, suggesting a shared risk factor for obesity and SCFE. Leptin is a cytokine-like hormone with proinflammatory properties known to be associated with autoimmune disorders, infections, and endocrine and metabolic diseases (Procaccini et al. 2015). Higher leptin levels with an effect on chondrocytes via interleukin (IL)-1 regulation were found in the serum and synovial fluid of patients with osteoarthritis and rheumatoid arthritis (Yan et al. 2018). However, an effect on the growth plate, if any, has not yet been investigated. Another pathway is the link between high leptin levels and thyroiditis, the most common cause of hypothyroidism (Procaccini et al. 2015) and the association of hypothyroidism with SCFE (Marquez et al. 2014, Uday et al. 2014, Kadowaki 2017). Case series and smaller retrospective studies in patients with SCFE (Loder et al. 1995) have demonstrated abnormal levels of thyroid hormone typical for hypothyroidism, but this study is the first to show a 3-fold higher risk of hypothyroidism in patients with SCFE compared with their matched controls in a population-based setting. The risk of hypothyroidism was almost 5-fold higher in male patients with SCFE and 2-fold higher in female patients with SCFE compared with their respective controls. The prevalence rate of hypothyroidism in Europe is between 0.2% and 5%, being higher in females (Chaker et al. 2017) with an estimated prevalence of undiagnosed hypothyroidism of 6% in females and 3% in males (Garmendia Madariaga et al. 2014).

Although SCFE is a childhood hip disease it can lead to hip pain and premature osteoarthritis of the hip in adulthood (Goodman et al. 1997), especially in patients who suffered severe slips, complications, or both (e.g., impaired range of motion of the hip joint, avascular necrosis of the femoral head or chondrolysis) (Tosounidis et al. 2010). The association between osteoarthritis and depression is well documented (Moussavi et al. 2007, Veronese et al. 2017) and the psychological burden of a chronic disease is not a novel concept (Moussavi et al. 2007). However, this study did not reveal a strong association of SCFE with depression, which could be explained by the assumption that pediatric patients cope better than adults with chronic diseases. Our cohort includes both mild and severe slips and severe slips presumably pose a higher risk of developing early osteoarthritis (Novais and Millis 2012). It is reasonable to suppose that the risk of depression might be higher in those who have severe slips and who later develop osteoarthritis than in those with mild slips.

This is the first study investigating the all-cause mortality in patients with SCFE. The 2-fold higher mortality risk together with an overrepresentation of vascular and endocrine causes of death in SCFE patients could be explained by the higher prevalence and risk of obesity and hypothyroidism. Flegal et al. (2013) found a higher risk of all-cause mortality in patients with severe obesity (BMI > 35). However, a lower mortality risk was found in individuals with overweight (BMI 25–< 30) or mild obesity (BMI 30–< 35). Hypothyroidism (even subclinical) is also associated with higher all-cause mortality (Huang et al. 2018). Another explanation for increased mortality risk relates to socioeconomic factors. Perry et al. (2017) found higher incidences of SCFE in individuals living in deprived areas. In addition, an association has been noted between lower socioeconomic status and higher mortality risk (Foster et al. 2018).

In most patients the comorbidity was diagnosed and registered after the diagnosis of SCFE. This could either be because the main focus was on the hip and not on the comorbidity or because of the fact that the comorbidity was not apparent at the time of the SCFE diagnosis. In Sweden, SCFE patients are treated at orthopedic departments and blood test for thyroid hormones is not routinely taken in patients with orthopedic diseases. Concerning obesity, the diagnosis might have been apparent at the same time at the SCFE diagnosis but not registered.

This study has a number of limitations. Unfortunately, I had no information on the socioeconomic background of our cohort. The study covers a long time period with parallel changes in ICD systems, and, additionally, changes in the coding practice may have influenced the results. This problem was addressed by analyzing the ICD periods separately, indicating no major differences in outcomes (data not shown). Furthermore, diagnoses that were managed in outpatient settings prior to 2001 have been missed, but this applies for both the patients and the control group, which makes a profound influence on the estimates attained unlikely. A separate analysis including only patients and control individuals diagnosed after 2001 was performed, but this resulted in no major differences in the attained risk estimates. Patients with diagnoses that can be treated in primary healthcare are not included in the Swedish Patient Register, which explains the low prevalence of obesity and depression in this study. The estimated prevalence of obesity in Sweden in 2011 was 14% in men and 13% in women aged 16–84 years (WHO 2013). Another drawback is that the Swedish Patient Register does not provide information on laterality or bilateral affection during the observation period. Bhatia et al. (2006) showed that patients bilaterally affected by SCFE had higher BMI than patients affected unilaterally. The prevalence of hypothyroidism and other endocrine disorders seems to be related to bilateral slips (Wells et al. 1993). Because patients with a history of SCFE inevitably have more frequent contacts with the healthcare system detection bias might be an issue. Nevertheless, in most patients there was a considerable time interval between the diagnosis of SCFE and the diagnosis of the comorbidity, which makes this scenario less likely. In addition, SCFE and its consequences, such as impingement of the hip joint or premature osteoarthritis, are mainly treated by orthopedic surgeons, whereas endocrine and metabolic diseases are often diagnosed by other specialists, which makes detection bias as an issue less likely. The higher risk for all-cause mortality in patients with a history of SCFE might also serve as an argument against a large impact of detection bias.

## Conclusions

For patients with SCFE and physicians it might be important to be aware of the higher lifetime risk of developing obesity, enabling preventive measures. Despite a rise in the prevalence of obesity in children and adolescents, the incidence of SCFE did not increase in Sweden over time. Patients with SCFE had a higher risk of hypothyroidism and a higher risk of all-cause mortality compared with a control cohort without SCFE. However, a cost–benefit analysis of screening for hypothyroidism needs to be performed before giving recommendations. The psychological burden of SCFE, as expressed in the risk of developing depression, was not confirmed in this study. However, the total burden of SCFE should be investigated more fully using patient-reported outcome measures.
